# Comparison of the Incorporation of DHA in Circulatory and Neural Tissue When Provided as Triacylglycerol (TAG), Monoacylglycerol (MAG) or Phospholipids (PL) Provides New Insight into Fatty Acid Bioavailability

**DOI:** 10.3390/nu10050620

**Published:** 2018-05-15

**Authors:** Frédéric Destaillats, Manuel Oliveira, Viktoria Bastic Schmid, Isabelle Masserey-Elmelegy, Francesca Giuffrida, Sagar K. Thakkar, Lénaïck Dupuis, Maria Laura Gosoniu, Cristina Cruz-Hernandez

**Affiliations:** 1 Nestlé Nutrition Product Technology Center, Rue Entre-Deux-Villes 10, CH-1814 La Tour-de-Peilz, Switzerland; Frederic.Destaillats@nestle.com; 2 Nestlé Research Center, Vers-chez-les-Blanc, CH-1000 Lausanne, Switzerland; manuel.oliveira@rdls.nestle.com (M.O.); viktoria.bastic@gmail.com (V.B.S.); isabelle.masserey-elmelegy@rdls.nestle.com (I.M.-E.); francesca.giuffrida@rdls.nestle.com (F.G.); Sagar.Thakkar@rdls.nestle.com (S.K.T.); Lenaick.Dupuis@rdls.nestle.com (L.D.); MariaLaura.Gosoniu@rdls.nestle.com (M.L.G.)

**Keywords:** docosahexaenoic acid, phospholipids, long-chain polyunsaturated fatty acids, *sn*-1(3)-monoacylglycerol, triacylglycerol

## Abstract

Phospholipids (PL) or partial acylglycerols such as *sn*-1(3)-monoacylglycerol (MAG) are potent dietary carriers of long-chain polyunsaturated fatty acids (LC-PUFA) and have been reported to provide superior bioavailability when compared to conventional triacylglycerol (TAG). The main objective of the present study was to compare the incorporation of docosahexaenoic acid (DHA) in plasma, erythrocytes, retina and brain tissues in adult rats when provided as PL (PL-DHA) and MAG (MAG-DHA). Conventional dietary DHA oil containing TAG (TAG-DHA) as well as control chow diet were used to evaluate the potency of the two alternative DHA carriers over a 60-day feeding period. Fatty acid profiles were determined in erythrocytes and plasma lipids at time 0, 7, 14, 28, 35 and 49 days of the experimental period and in retina, cortex, hypothalamus, and hippocampus at 60 days. The assessment of the longitudinal evolution of DHA in erythrocyte and plasma lipids suggest that PL-DHA and MAG-DHA are efficient carriers of dietary DHA when compared to conventional DHA oil (TAG-DHA). Under these experimental conditions, both PL-DHA and MAG-DHA led to higher incorporations of DHA erythrocytes lipids compared to TAG-DHA group. After 60 days of supplementation, statistically significant increase in DHA level incorporated in neural tissues analyzed were observed in the DHA groups compared with the control. The mechanism explaining hypothetically the difference observed in circulatory lipids is discussed.

## 1. Introduction

Linoleic acid (LA) and α-linolenic acid (ALA) are considered essential and can be converted into long-chain polyunsaturated fatty acid (LC-PUFA) metabolites that are responsible for important biological processes [1,2]. In humans, the enzymatic conversion from ALA to n-3 LC-PUFA such as eicosapentaenoic acid (EPA) and docosahexaenoic acid (DHA) is ranging from 4 to less than 0.05% [3]. It is well recognized that n-3 LC-PUFA are essential for normal growth and development [4,5,6]. To maintain adequate levels, humans needs to source dietary n-3 fatty acids from animal foods and in preference from fish and marine products [7].

n-3 LC-PUFA are integral part of cell membranes and provide the substrates for the production of signaling molecules that regulate blood clotting, contraction and relaxation of arterial walls, and inflammation [7,8] and immune response [6]. Likely, due to these effects, n-3 LC-PUFA have been shown to help prevent heart disease and stroke, may also help to reduce eczema and rheumatoid arthritis, and play protective roles in other conditions such as cancer [9,10,11,12]. DHA is highly abundant in the brain and retina and it is important to ensure optimum neural and visual functions [13].

Most dietary lipids are present in foods as triacylglycerols (TAGs) or in small proportions as monoacylglycerol (MAG), diacylglycerol (DAG) or phospholipids (PL). TAGs are hydrolyzed by lipolytic enzymes into free fatty acids and *sn*-2-monoacylglycerols (*sn*-2-MAG) mainly by the *sn*-1,3 specific pancreatic lipases in the upper part of the gastrointestinal tract. Free fatty acids and *sn*-2-MAG are absorbed by enterocytes and re-esterified to form TAG, cholesteryl esters and PL (mainly phosphatidylcholine), which are assembled into chylomicrons and secreted into lymphatic circulation. The metabolic fates and hydrolysis rates depend on the chain length, nature of the fatty acid in the TAG molecule and stereospecific location on the TAG. Indeed, there is evidence that the positional specificity has an effect on the uptake and impact of particular FA bioavailability [1,14,15,16]. 

Ensuring optimal absorption of n-3 LC-PUFA by optimizing their galenic form has become an important part of nutritional and therapeutic strategies. As a consequence, numerous studies have assessed the absorption, bioavailability and accretion into various tissues of different forms of n-3 LC-PUFA (mainly EPA and DHA) such as structured TAG, PL, free fatty acids, ethyl esters (EE), MAG, and DAG [14,15,17,18,19,20]. Intestinal absorption of DHA and EPA given as EE was reported to be lower than when provided as TAG or free fatty acids and different results were reported when related to lipoprotein studies [21,22,23,24]. Others report differences in the absorption of DHA and EPA when either esterified at the *sn*-2 or at the *sn*-1,3 positions of TAG [14,15,18,25,26]. We reported MAG are potent dietary carriers of DHA and EPA by providing superior bioavailability under impaired absorption conditions compared to conventional dietary TAG [19,27].

However, the absorption and incorporation of DHA in tissues when provided as PL-DHA and MAG-DHA have never been compared to TAG-DHA in a single design experiment. In the present study, the incorporation of DHA in circulatory lipids (plasma and erythrocytes) has been investigated longitudinally over 60 days. Retina and brain region tissues were also analyzed to assess the benefit of using such alternative DHA carriers on DHA accretion in these tissues.

## 2. Materials and Methods

### 2.1. Animals and Experimental Diets

The sample size, age and type of animal were selected based on previous experiment on effect of dietary carriers of LC-PUFA in a rat model [19]. All experimental procedures involving animals were approved by the cantonal veterinary office in Switzerland (reference: 2330VD) and reviewed by internal ethical committee. Six-week-old male Wistar rats (*n* = 80, 190 ± 11 g) were included and housed in independent cages (Euro standard Type III H: 425 × 266 × 185 mm-floor area 800 cm^2^, Techniplast, Gams, Switzerland), received tap water and diets ad libitum for 60 days. Animals were randomly divided into four groups. 

The lipid composition of the control diet consisted of AIN 93M base diet supplemented with sunflower oil, soybean oil (Sofinol S.A., Manno, Switzerland) and cocoa butter (Gerkens Cacao^®^, EJ Deventer, The Netherlands) as lipid source. Total lipid content was 20 g/100 g diet (approximately 40% of the energy, Table 1). Diets TAG-DHA, MAG-DHA and PL-DHA were AIN 93M base diet supplemented respectively with DHA oil (TAG-DHA group, DHASCO oil, Martek Biosciences Corporation, Columbia, MD, USA); *sn*-1(3)-MAG-DHA (MAG-DHA group, Cognis GmbH, Illertissen, Germany), PL-DHA (GPL8-DHA group, ASL, Hauterive, France), while control diet did not contain any source of DHA (Table 1).

The homogenized ingredients were converted to pellets, dried at low temperature and stored in small sachets under vacuum at −20 °C. These special precautions were taken to avoid oxidative degradation of n-3 LC-PUFA. All animals were fed fresh pellets every day. Similar levels of DHA in MAG-DHA groups were achieved (Table 2) while the incorporation of DHASCO oil in the TAG-DHA experimental diets resulted in a slightly lower level compared to the other DHA groups (0.29% of dry matter weight vs. 0.31% and 0.34% for the MAG-DHA and PL-DHA groups, respectively; see Table 2).

### 2.2. Experimental Design

Body weight of each animal was recorded twice a week for 60 days. Food intake was recorded five times a week. Body composition (fat and lean mass) was determined in animals, three measurements per animal, by quantitative nuclear magnetic resonance spectroscopy (Echo MRI-4in 1/500™; Echo Medical Systems, Houston, TX, USA), before the diet intervention (day 0) and the necropsy days (day 60).

Blood was collected into heparinized tubes from the caudal vein after 6 h of food restriction at days 0, 7, 14, 28, 35 and 49. The day of the necropsy at day 28 (*n* = 10/group) and day 60 (*n* = 10/group), animals were anesthetized with isoflurane where blood was collected from the abdominal aorta. After collection, blood was centrifuged (626× *g* for 10 min) and prepared as previously described [28]. Blood tubes were always kept on ice and erythrocytes and plasma sampled as follows: 200 µL of plasma were added into a 10 mL screw cap glass test tube containing 200 µL of ethanol; 200 µL of erythrocytes were added into a 10 mL screw cap glass test tube containing 200 µL of a erythrocyte lysis buffer. Buffy coat was discarded before sampling the erythrocytes. The tubes were equipped with Teflon-lined screw caps perfectly fitting the test tubes. Both tubes for plasma and erythrocytes were vortexed for 10 s before storage at −80 °C for further lipid analysis.

Retina, hippocampus, hypothalamus, and cortex were sampled and transferred to different vials, flash frozen with liquid nitrogen and stored at −80 °C until further analyses.

### 2.3. Sample Preparation

Brain (hippocampus, hypothalamus, and cortex) and retina analysis has been described in detail elsewhere [29]. Briefly, frozen brain tissues stored at −80 °C were placed in a dry iced cold mortar and further pulverized using liquid nitrogen to avoid any lipase action. Pulverized samples (50 mg) were placed in 10 mL screw cap tubes for immediate preparation of fatty acid methyl esters (FAME) as described in the next section. Before methylation, retina was homogenized in methanol (2 mL) with a Polytron homogenizer (Polytron PT 1300D; Luzern, Switzerland) for 2 min.

### 2.4. Fatty acid Methyl Esters Preparation and Analysis by Gas Chromatography

Preparation of FAME from erythrocytes and plasma was performed as previously described [28]. FAME were prepared from erythrocytes directly in the tubes in which they were sampled and stored. The internal standards 21:0 FAME (1 mg/mL) and 23:0 PC (1,2-ditricosanoyl-sn-glycero-3-phosphocholine, 0.2 mg/mL) were added (100 µL each) to erythrocytes, retina and brain samples. After, methanol was added to the tube (2 mL) followed by the addition of 2 mL 3 N solution of HCl gas in anhydrous methanol (wt/vol; Supelco Inc., Bellefonte, PA, USA) and finally 1 mL of n-hexane. Test tubes were firmly capped, shaken vigorously and heated at 100 °C for 90 min, with occasional shaking. After cooling down, water was added (2 mL) and the n-hexane phase was subjected to GC analysis after separation (1200 g for 5 min). The same procedure was followed for plasma samples except that 23:0 PC was substituted by 100 µL of internal standard 13:0 TAG and the transesterification was performed at 100 °C for 60 min. Similar methylation procedure was used to quantify fatty acid content in diets. Briefly, experimental diet (50 mg) was mixed with the catalyst methanol/HCl, 3N (2 mL), methanol (2 mL) and n-hexane (1 mL) to a test tube. The 21:0 FAME and 13: TAG (100 µL, 0.5 mg/mL) were used as internal standards.

Analysis of total FAMEs was performed on a 7890 Agilent gas chromatograph (Agilent Technologies, Palo Alto, CA, USA), equipped with a fused-silica BPX-70 capillary column (10 m × 0.1 mm I.D., 0.2 µm film thickness; SGE, Melbourne, Australia). Split injector (50:1) and flame ionization detection (FID) systems were operating at 250 °C. Oven temperature programming was 50 °C isothermal for 0.2 min, increased to 180 °C at 120 °C /min, isothermal for 1 min at this temperature then increased to 220 °C at 20 °C/min and then to 250 °C at 50 °C/min. The carrier gas (H2) was maintained at 1 mL/min and the acquisition of the FID signal at 100 Hz [30].

### 2.5. Statistical Analyses

Parameters are described per group by sample size (n), mean and standard deviation (SD). Outcomes were analyzed using parametric methods. Change score from baseline (day 0) was analyzed for outcomes that were measured at baseline (ten animals per group). In particular, the effects of each diet on the change from baseline in body weight gain and body composition were assessed using ANCOVA, adjusting for baseline measurements and daily food intake. The incorporation of DHA in erythrocytes and plasma were analyzed as change from baseline, using a linear mixed model with diet group, baseline measurements and daily food intake as fixed effects and subject-level random effect to take into account the longitudinal design. DHA levels in retina and brain tissues were analyzed using ANCOVA, adjusted for diet group and daily food intake (no adjustment for baseline was possible because of unavailable data at baseline). *p*-values lower than 0.05 were considered statistically significant. All statistical analyses were performed using R 3.1.2 (R core Team, Vienna, Austria).

## 3. Results

### 3.1. Food Intake, Body Weight Gain and Body Composition

Food intake was similar for all groups when compared with control group except for the PL-DHA group which was significantly higher (+11%, *p* = 0.009 compared to the control group Table 3). As expected, a progressive increase in body weight was observed for all groups along the study period and no difference between groups was observed at day 60 (Table 3). Body composition parameters were measured at baseline and at the end of the experimental period. No significant differences were found for fat mass or lean mass at baseline. There were no statistically significant changes in fat mass or lean mass after 60 days between any of the DHA diets and the control (Table 3).

### 3.2. Incorporation of DHA in Erythrocytes, Plasma, Retina and Brain Tissues

As we observed differences in food intake between groups (Table 3), changes from baseline were analyzed using a linear mixed model with diet group, baseline measurements and daily food intake as fixed effects and subject-level random effect to take into account the intra-subject correlation.

Incorporation of DHA in erythrocytes at different time points are shown in Table 4 for all treatments. As expected, the level of DHA in erythrocytes did not evolve in animals fed the control diet while it increased for all DHA groups compared to baseline. The incorporation of DHA in erythrocytes for the animals fed with TAG-DHA compared with the control group was statistically significant higher at all time-points. Similarly, statistically significant increase in the incorporation of DHA in erythrocytes were observed for animals fed with PL-DHA and MAG-DHA compared with the control group at all time-points. The comparisons between PL-DHA and MAG-DHA diets versus the TAG-DHA diet showed statistically significant increase in the PL-DHA for all time points and in the MAG-DHA diet for day 28, 35 and 49 (Table 4). Furthermore, the comparison between animals fed with PL-DHA and MAG-DHA diets shown a statistically higher concentration of DHA in animals fed with PL-DHA diet at day 14 and 49.

In plasma, the DHA level was similar at baseline for the DHA diet groups when compared with control group. As observed in erythrocytes, the incorporation of DHA in plasma lipids significantly increased during the experiments for all DHA fed groups and all time points when compared with control group (Table 5). The level of DHA in plasma was statistically significant higher in the PL-DHA group compared with the TAG-DHA group, at day 14, 35 and 49 (Table 5). The comparisons between MAG-DHA and TAG-DHA groups did not show any statistically significant differences. DHA levels measured in plasma of animals fed with PL-DHA diet were statistically higher compared to animal fed with MAG-DHA at day 14 and 49 (Table 5).

Levels of DHA in retina, cortex, hypothalamus and hippocampus brain regions are shown in Table 6. In all DHA fed groups, the DHA levels measured at 60 days were statistically significant higher when compared with control group, except for PL-DHA group in hypothalamus. No significant difference was observed in the level of DHA in retina between the MAG-DHA and PL-DHA groups.

## 4. Discussion

**Figure 1:**
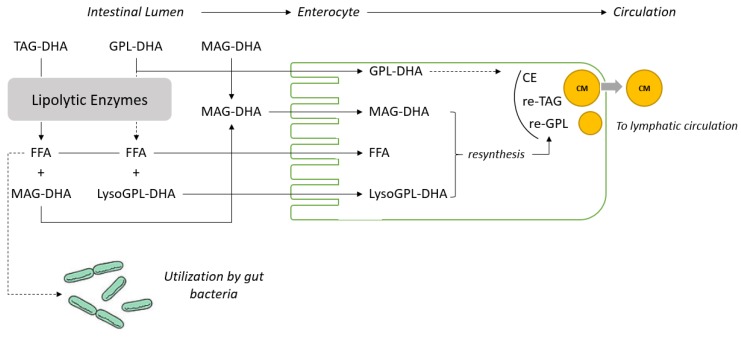
Schematic representation of hypothetical pathways for the digestion, absorption and secretion in the circulation of DHA provided as triacylglycerol (TAG-DHA), phospholipids (PL-DHA), and *sn*-1 (3)-monoacylglycerol (MAG-DHA). Abbreviations used: LysoPL-DHA, lyso-phospholipids esterified with DHA; FFA, free fatty acid; CE, cholesteryl ester; re-TAG, TAG resynthesized in the enterocyte; re-PL, PL resynthesized in the enterocyte and CM, chylomicron. The dashed arrows correspond to the hypothesis discussed.

During the 8-week study under the experimental diets all animals grew as expected. The difference observed in food intake in the PL-DHA group was reflected in the body weight gain (Table 3). To adjust for the difference in food intake, a parametric statistical model has been used. In addition, the accretion of DHA in different important organs such as adipose tissue was not measured and circulatory lipids (erythrocytes and plasma) were considered in the present study as surrogate markers to assess the potency of the different dietary DHA carriers. Nevertheless, the results obtained for the TAG-DHA group in this study and the literature available on the digestion, absorption and metabolism of TAG, MAG and PL give good insight to understand the fate of the fatty acids provided by these different dietary carriers as represented in Figure 1.

**Figure 2:**
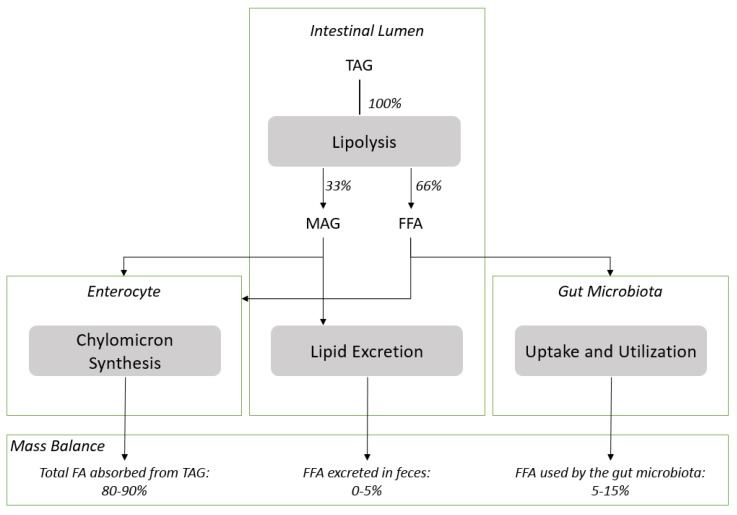
Schematic representation of the hypothetical fate of fatty acids during the digestion of dietary TAG and mass balance distribution estimated among the three biological compartments. The estimation is based on data obtained in present study and by others on DHA or other LC-PUFA as model fatty acids provided as TAG and alternatives carriers such as MAG, which are absorbed quantitatively. Abbreviations used: TAG, triacylglycerol; FFA, free fatty acid; MAG, monoacylglycerol.

We previously demonstrated in animal and human model conditions using lipase inhibitors that n-3 LC-PUFAs as *sn*-1(3)-MAG can be directly absorbed by enterocytes and do not require the action of pancreatic lipases [19,27]. This hypothesis is further supported by the data obtained using DHA recovered from lymphatic cannulated rats fed with TAG-DHA, DAG-DHA, MAG-DHA and EE-DHA [20]. Analysis of the lymphatic recovery of DHA from TAG-DHA, DAG-DHA, MAG-DHA and EE-DHA confirmed that the level of DHA absorbed is much higher when provided as MAG [20]. It can therefore be concluded that MAG are channeled very efficiently from the intestinal lumen to the enterocyte as displayed in Figure 2. In the present study, significantly higher levels of DHA in erythrocyte lipids were observed in animals fed with MAG-DHA and PL-DHA compared with animals fed with TAG-DHA (Table 4). These results strongly suggest that PL-DHA enriched oil may also require minimal digestion prior to absorption (Figure 1).

The evolution of the concentration of DHA, for all groups, in plasma and erythrocytes is consistent with data reported elsewhere showing that dietary supplementation with DHA leads to a progressive increase of the concentration of DHA in circulatory lipids until it reaches a steady state concentration [1]. It has been demonstrated that the DHA level reached at steady states in circulatory lipids depends on the concentration of DHA in the diet [1]. The differences observed in circulatory lipids were larger than the differences in concentration of DHA in the diets suggesting that the level reached at steady state depends not only on the level of DHA supplemented but also on the nature of the dietary carrier used. To explain such differences, it is necessary to revisit the basics of the digestion of dietary lipids such as TAG and take in consideration recent progress in understanding the lipid metabolism of the gut microbiota.

TAG are the main source of dietary lipids and TAG digestion by pancreatic lipase and colipase requires the emulsification of TAG in the presence of bile salts to generate the aqueous interface required for the enzyme to function [17]. The process of emulsification is triggered by the moderate hydrolysis of bile PL that is catalyzed by pancreatic phospholipase A2 [31]. The Lyso-PL formed from bile PL are facilitating the emulsification process of the dietary TAG. However, it does not imply that dietary PL need to be quantitatively hydrolyzed before being absorbed as PL can spontaneously form micelles due to their amphiphilic nature. It has been reported that fatty acids linked to Lyso-PL are preferentially incorporated and channeled by the HDL [32]. MAG and free fatty acids in a 1:2 ratio formed by the partial lipolysis of dietary TAG are absorbed and re-esterified to form lipid droplets that are excreted as chylomicrons into the lymphatic system (see Figure 2 for schematic representation). The level of absorption of dietary lipids is commonly measured using the so called “apparent digestibility” method which is calculated as follow: [(lipid intake–fatty acid excretion in feces)/(fatty acid intake) × 100]. The results obtained under physiological conditions with this method are extremely high (>95%) and suggest that the absorption of the lipolytic products formed during the digestion of dietary TAG is quantitative. The apparent digestibility can be lowered under maldigestion conditions induced for example by inhibition of the lipase function or lipid malabsorption. In these conditions, lipids are excreted with the consecutive apparition of steatorrhea [29]. The apparent digestibility of n-3 LC-PUFA from fish oil (TAG) and krill oil (PL) was compared in rat models and results were found to be identical [33] while the n-3 LC-PUFA deposition was higher in animals fed with the krill oil diet. Others reported that incorporation in infant piglets of DHA from PL-DHA in plasma phospholipids as provided by egg phospholipids was significantly higher (c.a. 50%) than TAG-DHA provided by fungal oil [34]. In accordance with literature data, we found that the incorporation of DHA in plasma and erythrocyte lipids was higher with MAG-DHA and PL-DHA compared to TAG-DHA, even if corrected for the bias related to the level of DHA provided by the TAG-DHA diet (Table 4 and Table 5). Therefore, if the apparent digestibility data reported in literature are similar for PL-DHA and TAG-DHA but their level of circulatory lipids differs, it suggests that part of the free fatty acids released from TAG are not absorbed nor excreted.

Gut microbiota “metabolism” is extremely active and the lipid metabolism of model gut bacteria such as *Escherichia coli* has been characterized in detail [35]. It is well established that *E. coli* is able to use various type of fatty acids as a source of energy [35]. *E. coli* can uptake free fatty acids from the intestinal lumen and transport them into the cytosol via a transport/acyl-activation mechanism involving an outer membrane protein, FadL, and inner membrane-associated FadD [acyl-CoA synthase (fatty acid-CoA ligase)]. Fatty acids that are absorbed by *E. coli* are beta-oxidized resulting in the formation of acetyl coA [35]. It seems that other bacteria such as *Lactobacillaceae* are able to metabolize fatty acids as well [36]. Therefore, we can hypothesize that part of the free fatty acids released from TAG during the hydrolysis process catalyzed by the lipase and colipase can be up-taken and utilized by the gut microbiota as schematically represented in Figure 1.

It can be estimated that the gut microbiota utilize 5–15% of free fatty acids released from TAG as represented; assuming that the quantification of DHA in enterocyte and plasma lipids is representative of the DHA level absorbed and that the level of free fatty acids excreted in feces under physiological condition is lower than 5% (Figure 2). Precise evaluation of the level of utilization of free fatty acids released from the digestion of lipids by gut microbiota needs to be further demonstrated in dedicated experiments. However, it suggests that the assessment of the “apparent digestibility”, which provides in most of the cases a very high value, might not provide a quantitative estimation of the dietary fatty acid absorbed.

Together with bioavailability, several reports have documented different efficacy and incorporation of DHA in tissues after feeding PL and TAG [33,34,37,38]. DHA supplementation has shown effects on attention-deficit/hyperactivity disorders and hyperactivity in children [39,40]. Higher levels of DHA in brain tissue accretion were found in term baboons [41] and mice [42] when provided as PL compared to TAG. Most studies have examined the alteration of fatty acid composition by analyzing the whole brain. However, specific differences in phosphoglycerides in neonatal rats or mice have been only identified in certain brain regions such as frontal cortex and hippocampus [43,44,45]. Thus, it appeared more informative to investigate the fatty acid composition of specific brain regions in order to establish relationships with the studied conditions. Three brain regions were sampled in this study: cortex, hippocampus and hypothalamus with the aim to establish the fatty acids composition of each region with the three different DHA carriers supplemented. The region with the highest DHA levels was the cortex, followed closely by the retina.

These results agree with those observed in rats by [43,44,45] who found more DHA in frontal cortex than in the striatum. Similar increments were observed for all DHA treatments when compared with control. Plasma fatty acids content has been highly related to tissue fatty acids status and cognitive functions [46,47], which is reflected in the results we obtained in retina, hippocampus and hypothalamus. In contrast with plasma and erythrocyte lipids, the level of DHA in neural tissues of adult animals is high and it seems that it cannot be increased in a dose dependent manner over a certain level. Therefore, we did not observe major differences in the level of DHA found in these neural tissues when using different dietary carrier of DHA after 60 days of treatment (see Table 6). However, testing the hypothesis under different conditions such as depleted adults or neonatal animal models might provide interesting insight on the impact of the dietary carriers of DHA on the incorporation in DHA in neural tissues as previously observed for arachidonic acid [41].

## 5. Conclusions

In this study, the bioavailability of different DHA derivatives was assessed using circulatory lipids as markers; deposition of DHA in tissues was not considered with the exception of retina and selected brain regions. The conclusion related to the bioavailability is therefore limited to the biological compartments analyzed. The experimental data obtained suggests that partial acylglycerols such as MAG-DHA or PL-DHA are more efficient than TAG-DHA to increase DHA level in erythrocytes. Assuming that the difference observed is directly related to absorption, it might indicate that part of the free fatty acids generated during the digestion of TAG is utilized as a carbon source by the microbiota and the remaining portion secreted in feces.

## Figures and Tables

**Table 1:** Composition of the experimental diets (in % of dry matter weight).

Composition	Control	TAG-DHA	MAG-DHA	PL-DHA
Total lipid content	20.00	20.00	20.00	20.00
Cocoa butter	3.00	1.60	2.20	1.70
Soybean oil	11.00	11.20	11.00	10.00
Sunflower oil	6.00	6.46	6.00	5.20
Oil DHASCO (TAG-DHA)		0.76		
*sn*-1(3)-Monoacylglycerol (MAG-DHA)			0.74	
GPL8-DHA (PL-DHA)				3.10
Corn starch	46.10	46.10	46.10	46.10
κ-Caseinate	14.00	14.00	14.00	14.00
Sucrose	10.00	10.00	10.00	10.00
Cellulose	5.00	5.00	5.00	5.00
Mineral mix AIN-93M	3.50	3.50	3.50	3.50
Vitamin mix AIN 93VX	1.00	1.00	1.00	1.00
l-cystine	0.18	0.18	0.18	0.18
Choline bitartrate	0.25	0.25	0.25	0.25
Butylhydroxytoluene (BHT)	0.0008	0.0008	0.0008	0.0008

**Table 2:** Fatty acid composition of the experimental diets (in % of dry matter weight).

Composition	Control	TAG-DHA	MAG-DHA	PL-DHA
14:0	0.02	0.08	0.05	0.02
16:0	1.81	2.02	2.09	2.16
16:1 n-7	0.02	0.03	0.03	0.04
17:0	0.04	0.04	0.04	0.04
18:0	1.44	1.61	1.66	1.17
18:1 n-9	4.52	5.10	5.16	4.64
18:2 n-6	6.87	7.47	7.59	6.95
18:3 n-3	0.48	0.52	0.53	0.47
20:0	0.04	0.08	0.08	0.06
20:1 n-9	0.03	0.04	0.03	0.04
20:5 n-3	0.08	0.09	0.09	0.08
22:1 n-9	0.00	0.00	0.00	0.00
22:6 n-3	0.00	0.29	0.31	0.34
24:0	0.03	0.04	0.04	0.03
Total n-3 FA	0.56	0.90	0.93	0.90

**Table 3:** Anthropometry and dietary intake of rats fed control and experimental diets (mean ± SD).

Parameter		Experimental Group		
	Control	TAG-DHA	MAG-DHA	PL-DHA
Final body weight (g)	388.1 ± 13.4	398.8 ± 28.5	395.9 ± 23.6	421.04 ± 39.1
Fat Mass d 0 (g)	21.2 ± 3.6	21.8 ± 3.9	21.6 ± 3.3	22.2 ± 3.3
d 60 (g)	65.1 ± 12.7	77.5 ± 13.6	65.5 ± 14.5	79.7 ± 28.7
Lean Mass d 0 (g)	146.3 ± 7.9	146.5 ± 5.8	146.7 ± 6.9	145.8 ± 6.4
d 60 (g)	272.7 ± 16.7	270.6 ± 21.5	280.1 ± 13.7	288.0 ± 12.7
Daily Food Intake (g/d)	27.5 ± 10.1	25.3 ± 8.8	27.5 ± 7.2	30.6 ± 10.1 *

TAG-DHA = triacylglycerol DHA, MAG-DHA = monoacylglycerol MAG, PL-DHA= phospholipid DHA. Values represent means ± SD (*n* = 10). For statistics, data for groups fed diets supplemented with DHA have been compared to the group receiving control diet using an ANCOVA model, adjusted for baseline values and daily food intake. * *p* value lower than 0.05.

**Table 4:** DHA level in erythrocytes expressed in µg per ml at different time points (Mean ± SD).

Group	Feeding Time (days)				
	0	14	28	35	49
Control	44.2 ± 12.2	40.7 ± 6.0	51.0 ± 11.4	41.4 ± 10.0	54.2 ± 30.2
TAG-DHA	46.1 ± 14.0	58.2 ± 12.1 **	78.5 ± 13.8 ***	71.5 ± 15.4 ***	78.2 ± 14.9 ***
PL-DHA	42.3 ± 15.9	77.6 ± 17.4 ***^,§,!^	99.8 ± 19.2 ***^,!^	103.1 ± 15.5 ***^,!^	138.4 ± 71.4 ***^,§,!^
MAG-DHA	52.3 ± 9.7	64.4 ± 11.0 ***	98.8 ± 22.3 ***^,!^	94.1 ± 17.7 ***^,!^	105.8 ± 22.3 ***^,!^

**, and *** indicate *p* value lower than 0.05, 0.01 and 0.001, respectively (linear mixed model based P value, with adjustment for baseline and daily food intake values) when interventions group has been compared with control group. ^!^ Indicates *p* value lower than 0.05 when PL and MAG-DHA groups has been compared with TAG-DHA. ^§^ Indicates *p* value lower than 0.05 when PL-DHA group has been compared with MAG-DHA group.

**Table 5:** DHA level in plasma expressed in µg per mL at different time points (Mean ± SD).

Group	Feeding Time (days)				
	0	14	28	35	49
Control	42.1 ± 21.6	34.0 ± 7.9	29.7 ± 9.4	26.4 ± 8.6	32.2 ± 10.3
TAG-DHA	32.4 ± 15.4	54.5 ± 16.5 ***	48.9 ± 20.3 ***	59.9 ± 21.0 ***	75.3 ± 26.8 ***
PL-DHA	36.0 ± 11.8	102.2 ± 25.9 ***^,^^§,!^	68.8 ± 35.9 ***	92.6 ± 14.7 ***^,!^	102.7 ± 27.0 ***^,^^§^^,!^
MAG-DHA	48.7 ± 14.6	66.5 ± 19.3 ***	56.7 ± 17.8 ***	73.6 ± 18.4 ***	80.1 ± 22.5 ***

*** indicate *p* value lower than 0.05, 0.01 and 0.001, respectively (linear mixed model based P value, adjusted for baseline and daily food intake values) when interventions group has been compared with control group. ^!^ Indicates *p* value lower than 0.05 when PL and MAG-DHA groups has been compared with TAG-DHA. ^§^ Indicates *p* value lower than 0.05 when PL-DHA group has been compared with MAG-DHA group.

**Table 6:** Level of DHA in retina and brain tissue lipids in adult rats fed during 60 days with chow diet or different dietary carriers of DHA (in % of total fatty acids).

Tissue	Control	TAG-DHA	MAG-DHA	PL-DHA
Retina	28.92 ± 2.7	32.84 ± 1.2 ***	32.32 ± 1.4 ***	32.77 ± 1.3 ***
Cortex	12.75 ± 0.6	13.56 ± 0.7 ***	13.41 ± 0.4 ***	13.62 ± 0.5 ***
Hypothalamus	10.65 ± 0.7	11.29 ± 0.8 **	11.27 ± 0.4 **	10.98 ± 0.5
Hippocampus	10.96 ± 1.3	11.89 ± 1.0 **	12.34 ± 0.7 ***	12.42 ± 0.9 ***

**, and *** indicate *p* value lower than 0.05, 0.01 and 0.001, respectively (ANCOVA adjusted for baseline values and daily food intake) when interventions group has been compared with control group.
